# 

**Published:** 1886-06

**Authors:** 


### CONTENTS.

ORIGINAL COMMUNICATIONS—Hæmorrhage Before Delivery—Dr. Otto Betz, Heilbron, Germany....... 429
Differentiations of Diphtheria and Croup, Dr. W. A. Morris, Austin .............................. 530
Rupture of the Urinary Bladder, Dr. D. Berry, San Antonio .............................. 533
A Rare Case of Gunshot Wound, with Recovery, Dr. C. K. Gregg, Ramos Arespe, Mexico........ 536
Placenta Prævia Complicated with Hydatiform Cysts, Dr. R. H. L. Bibb, Saltillo, Mexico .... 537
Malarial Irritation of the Female Bladder, Dr. H. K. Leake, Dallas .............................. 540
A Two-Pouud Child, Dr. S. L. Post, Alvarado.... 543
CORRESPONDENCE—Lusus Naturæ, Letter from Dr. R. S. Gregg, Manor.............................. 545
Surgery in San Antonio, Letter from Dr. R. Menger 546
EDITORIAL—Dr. Austin Flint, and his Address in Medicine .............................. 549
Splenetic—Let the Galled Jade Wince.......... 551
Another Stultification, a Homœopathic Strike.... 553
The Round-Up—Prize Essay.................. 554–556
EDITORIAL NOTES—Practicing the Same—Yellow Fever Commission—As Through a Smoked Glass—darkly 556
BIBLIOGRAPHY.............................. 560
ADVERTISERS' NOTICES.............................. 568

